# Supraperiosteal technique protocol for forehead filling with a mixture of calcium hydroxyapatite and hyaluronic acid: Double‐blind, randomized controlled clinical trial

**DOI:** 10.1111/jocd.16488

**Published:** 2024-08-04

**Authors:** Natália Scardua, Denize Peruzzo Rovaris, Kelly Maria Silva Moreira, André Luiz Sena Guimarães, Maria Tereza Scardua

**Affiliations:** ^1^ Tereza Scardua Institute São Paulo São Paulo Brazil; ^2^ Department of Dentistry Universidade Estadual de Montes Claros (Unimontes) Montes Claros Minas Gerais Brazil

**Keywords:** aging, calcium hydroxyapatite, forehead filler, hyaluronic acid

## Abstract

**Objective:**

The current study aims to investigate the safety and efficacy of using calcium hydroxyapatite (CaHA) versus CaHA associated with hyaluronic acid (HA) for forehead volume replacement and contour restoration without forehead irregularities.

**Methods:**

This interventional study involved 132 participants in a two‐arm, parallel, double‐blind trial for forehead treatment using the supraperiosteal technique. Group A received CaHA, and Group B received a combination of CaHA and HA as filler materials. Follow‐up assessments occurred at 30 and 180 days, incorporating the 5‐point Global Aesthetic Improvement Scale (GAIS) and photographic analysis for forehead volume replacement, contour restoration, and without forehead irregularities. Safety assessments included monitoring adverse events, particularly nodules.

**Results:**

The study included all 132 enrolled patients who completed the trial. Applying CaHA in combination with HA resulted in a statistically significant improvement in both GAIS scale scores and the reduction of forehead irregularities. The total incidence of nodules was 3.7%. Group A had four times more occurrences of nodules than Group B. Furthermore, Group B exhibited lower rates of forehead irregularities following the treatment compared to Group A.

**Conclusion:**

The supraperiosteal application of CaHA and HA for forehead treatment demonstrates superior efficacy in addressing signs of aging compared to the isolated use of CaHA.

## 1 INTRODUCTION

The forehead represents the upper third of the face and displays a convex curvature, a reflection of the underlying frontal bone. Both defects in the frontal bone and its reabsorption in aging can be responsible for unsatisfactory aesthetic appearance on the forehead.^1^, ^2^ Furthermore, the facial expression muscles of that region and the effect of tissue loss with aging may play an essential role in the transition from dynamic to static wrinkles, contributing to the appearance of upper facial aging.^3^

Tissue fillers are widely used to enable non‐surgical restructuring of skin and soft tissues. Its use has been accepted due to its relatively simple and quick application associated with safety and effectiveness,^4^, ^5^ and it can be an effective alternative in treating static wrinkles of facial soft tissues.^3^, ^6^ However, filling treatments of the frontal area have essential limitations that cause professionals to use only botulinum toxin for the aesthetic treatment of that region. One of the main concerns is the possibility of intravascular injections and retrograde embolisms that can cause some rare but devastating adverse effects, such as blindness and stroke.^7^, ^8^, ^9^, ^10^ Another limitation is the scarce fat under the skin, which narrows the dermal filler implantation space and makes camouflaging any irregularities complex.^9^

In response to these limitations, techniques with safer protocols, primarily related to blindness, have already been published.^11^, ^12^ However, they have used only calcium hydroxyapatite (CaHA) as a filler material, which may not promote the ideal results, and it is free of adverse effects since it is the filler of a region with a thin layer of subcutaneous tissue prone to irregularities and nodules.

Currently, CaHA is an alternative to hyaluronic acid (HA) for specific objectives such as volume and tissue structuration due to its physical properties (viscosity and elasticity), giving it a more remarkable ability to lift the tissues.^13^, ^14^, ^15^, ^16^ However, CaHA does not present an enzyme that enables its reversal, and this is a fundamental feature of a filler when injected into a region so thin that it requires adjustments. Another worrying factor is the possibility of nodules, inherent to biostimulators, which, in an area such as the forehead, increases the chance of nodule formation due to the small thickness of the subcutaneous tissue becoming very apparent, with significant aesthetic impact.

According to publications, the strategy to solve these problems includes injecting a tumescent solution with saline solution and anesthetic before applying the filler, promoting tissue hydrodistention and reducing pain. The tumescent solution creates a bag of space to distribute the filler and prevents possible vascular impairments.^11^, ^12^ The technique described here is also based on creating an area before the injection of the filler material to avoid possible intravascular injections. The association between CaHA and HA is commercially evaluable, highlighting the benefits of association.^17^ The complex viscosity (η*) is a measure of resistance to flow and is relevant for assessing the potential for filler migration. Materials with high η* are less likely to migrate, even in highly mobile anatomical areas, such as the forehead.^18^ Given the need to increase volume gain, our current study protocol combines HA with higher reticulation.

Therefore, this study aimed to assess the effectiveness and safety of forehead volume replacement and contour restoration, specifically addressing forehead irregularities that were not effectively resolved by previous botulinum toxin treatment. The dissection technique was utilized to fill the forehead using CaHA associated with HA as the filler material.

## 2 METHODS

### 2.1 Ethical approval

All performed procedures involving human participants were conducted following the ethical standards of the institutional and national research committees, the 1964 Helsinki Declaration, and its later amendments or comparable ethical standards. Ethical approval for this study (No. 58145022.2.0000.5374) was obtained from the Institutional Review Board. Data were collected from July 2022 to March 2023. All patients signed an individual informed consent.

### 2.2 Compliance with ethical standards

The authors declare that they have no conflicts of interest.

### 2.3 Study design

The present investigation is an interventional, two‐arm, parallel, and double‐blind trial. The study was reported according to Consolidated Standards of Reporting Trials guidelines.^19^

### 2.4 Sample size calculation

**FIGURE 1:**
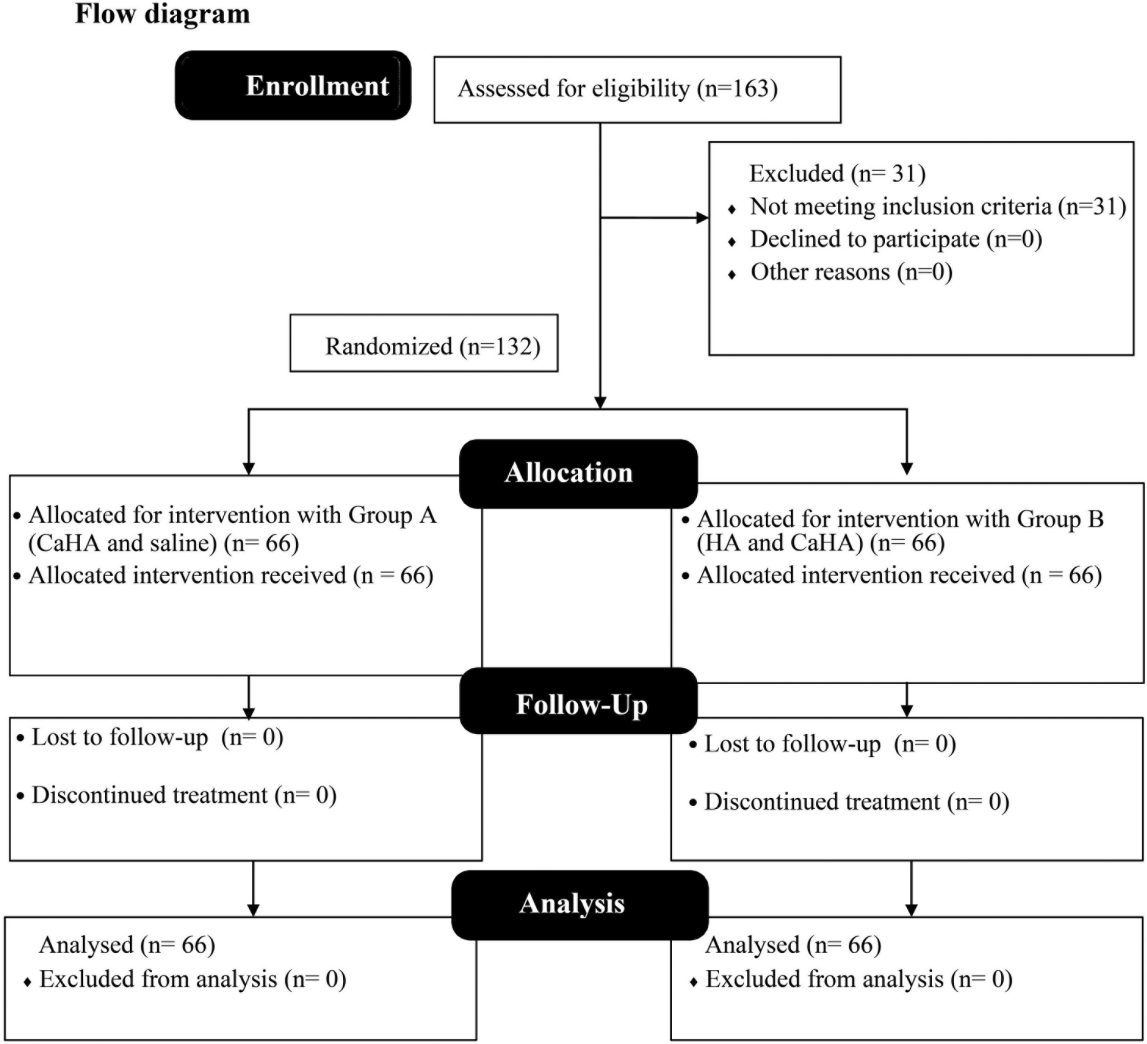
Study flow chart.

The sample calculation was based on the previous study.^20^ Global Aesthetics Improvement Scale (GAIS)^20^ the sample size calculation was performed to achieve an alpha of 0.05, a beta of 0.05, and a study power of 0.95. The sample size calculation used previously described parameters.^21^ According to the sample size test, at least 63 patients were necessary for each group. One hundred sixty‐three patients were assessed for eligibility, and 132 (66 and 66) were enrolled in the study. The flow diagram of the study is presented in Figure 1.

### 2.5 Allocation concealment and blinding

Before commencing the study, an independent researcher, uninvolved in treatment or recruitment, devised the plan for concealing allocation. A straightforward randomization sequence was generated through statistical software for allocation concealment. Another researcher then randomized patients into two groups, using a brown envelope to conceal the assignment to either option A or B, maintaining a 1:1 allocation ratio. Both patients and the professionals overseeing the assessment of forehead improvement were kept blinded to ensure impartiality in the evaluation process. Furthermore, the researchers responsible for statistical analysis and allocation differed from care providers or recruiters.

### 2.6 Inclusion criteria

The study included patients with forehead irregularities that were not effectively resolved by previous treatment with botulinum toxin despite the patients' complaints.

### 2.7 Exclusion criteria

Patients with a history of allergic reactions to fillers, individuals undergoing treatment with anticoagulants or immunosuppressants, those with autoimmune diseases, and pregnant or lactating women were excluded from the study. Participants who had received fillers or botulinum toxin for upper face enhancement within the preceding 6 months were also excluded to prevent interference with the initial assessment. Patients who underwent additional aesthetic treatments before the final evaluation were excluded from the study.

### 2.8 Intervention

In both groups, the previous application of botulinum toxin (Xeomin®–Merz Aesthetics, Frankfurt, Germany) was carried out in participants due to contraction of the frontal muscle 15 days before the filler. The doses varied according to muscle activity, between 6 and 20 U.

As a prophylactic medication for inflammation and edema, patients took one tablet of Predisin® (Mantecorp Farmasa, Anápolis, Goiás, Brazil) 1 h before the procedure—the supraperiosteal technique for forehead filler application strictly adhered to the previously described protocol.^22^ The area selected for the filling should comprise the central part of the forehead, where it usually presents more significant depression and volume loss. The lower limit is respected 2 cm above the orbital bone border because the supratrochlear artery and supraorbital artery are from this limit. Approximately 0.2 mL of 2% lidocaine with epinephrine is used for each anesthesia button on the sides of the delimited area, about 45 mm from the midline. Hence, the cannula reaches the entire region. The puncture was made using a 22G needle, which was sufficient to introduce a semi‐flexible disposable cannula (21G × 50 mm) to avoid bleeding and bruising. Subsequently, 1 mL of lidocaine with epinephrine was deposited along the entire length of the periosteum. After anesthetizing the area, the cannula is reinserted deeply to dissect all the tissue above the periosteum, thus creating a space to receive the filler material. The dissection procedure not only prevents embolisms but also facilitates the accommodation of the material. This detachment should be performed delicately to avoid injury to the periosteum and prevent significant post‐procedure edema. Treatment was carried out at the first consultation after obtaining clinical photographs, demographic information, and informed consent from patients. After treatment, patients took two subsequent follow‐up visits after 1 and 6 months.

### 2.9 Groups

#### 2.9.1 Group A (CaHA and saline)

For group A, which received only CaHA, 1.25 mL of Rennova® Diamond was diluted in 2.25 mL of saline solution. The diluted mixture was then transferred to a 5.0 mL sterile syringe using a female‐to‐luerlock connector. The two syringes were securely connected, and the materials were thoroughly mixed by alternately pressing the plungers until a homogeneous mixture was achieved. Subsequently, the diluted CaHA mixture was transferred to a 1 mL syringe and connected to a 21G × 50 mm Softfil microcannula (Soft Medical Aesthetics®, Paris, France) for application. The application process involved retrograde, slow injection, and simultaneous massage with each small quantity injected to ensure optimal distribution.

#### 2.9.2 Group B (HA and CaHA)

The study utilized Rennova Ultra Deep® (Panaxia LTD, Israel), a HA with relatively high viscoelasticity, for volumization. This HA was combined with CaHA (Panaxia LTD, Israel) to stimulate collagen production. The synthesis of collagen within the HA matrix aimed to provide enhanced tissue support and integration. The combination of CaHA with HA was chosen to promote volume while minimizing the risk of nodule formation. This combination offered partial reversibility and collagen stimulation.

The mixture comprised 1.25 mL of HA and 1.25 mL of CaHA, adding 1 mL of saline solution to facilitate material accommodation. This dilution reduced viscosity while maintaining elasticity and malleability. The preparation involved using two 5.0 mL sterile polypropylene syringes. Specifically, 1.25 mL of CaHA (Rennova® Diamond—Panaxia, Israel) and 1.25 mL of HA (Rennova® Ultra Deep Lido—Panaxia, Israel) along with 1 mL of saline solution were transferred to a 5.0‐mL sterile syringe using a female‐to‐luerlock connector. The two syringes were connected tightly, and the materials were mixed by alternately pressing the plungers until a homogeneous mixture was achieved. The diluted HA and CaHA were transferred to a 1‐mL syringe and connected to a 21G × 50 mm Softfil microcannula (Soft Medical Aesthetics®, Paris, France) for application. A 1‐mL syringe was chosen to ensure lower injection force and better material distribution. The application process was retrograde, slow, and involved concurrent massage with each small quantity injected to ensure optimal distribution.

### 2.10 Outcomes and measured parameters

The aesthetic improvement of the forehead was evaluated using the GAIS^20^, ^23^ of 5‐point, with scores of 1 (excellent), 2 (very good), 3 (good), 4 (unaltered), and 5 (worse), carried out in a double‐blind manner employing photographs obtained on the day of application, 30 and 180 days after the procedure. The pictures were analyzed by two trained and calibrated independent assessors (kappa intra e inter‐examinator de 0.81 e 0.85, respectively). In addition, the effectiveness of the treatment was evaluated by improving irregularities and the presence of nodules. No additional treatments were permitted until the final evaluation.

### 2.11 Statistical analysis

The data analysis was conducted using the Graph Pad Prism 8.0.1. The charts were performed using Microsoft Excel. The chi‐square and Fisher's exact tests were used to compare the two groups' irregularity and nodule presence data. A value of *p* ≤ 0.05 was considered statistically significant.

## 3 RESULTS

All 132 participants enrolled in the study successfully underwent 30‐ and 180‐day follow‐up assessments, as illustrated in Figure 1. Notably, there were no instances of participant dropout (Figure 1). Regarding demographic distribution, Group A comprised 23 males and 43 females, while Group B included 16 males and 40 females. Importantly, no statistically significant differences were observed in the characteristics of the patients between the two groups (Table 1).

**FIGURE 2:**
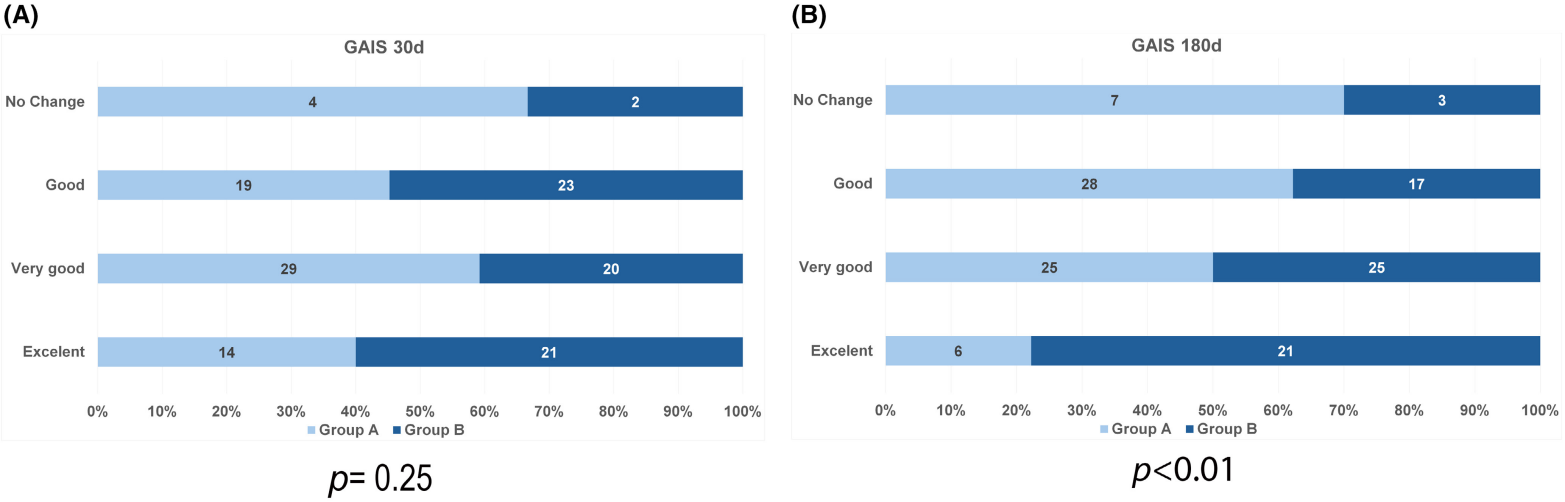
GAIS (Global Aesthetic Improvement Scale) scores at two‐time points: 30 days (A) and 180 days (B). Chi‐square tests were used to assess discrepancies between the groups. No significant differences were observed between Group A (CaHA and saline) and Group B (HA and CaHA) at the 30‐day assessment. However, at the 180‐day assessment, Group B exhibited a marginal increase in GAIS scores compared to Group A.

The chi‐square tests conducted for the study indicate that, after 30 days, Group B exhibited a marginal increase in GAIS scores compared to Group A, although this difference did not reach statistical significance (*p* = 0.25, as depicted in Figure 2). However, a noteworthy contrast emerges at the 180‐day mark, where Group B demonstrated significant enhancements in forehead aesthetics compared to Group A (*p* < 0.01, Figure 2). It is pertinent to highlight that the effect size determined by the chi‐square tests (12.62, data not shown) is 0.496, denoting a substantial magnitude. This effect size is categorically deemed significant, underscoring our study's robust impact. Notably, after 180 days, it becomes evident that Group B consistently displayed a progressive increase in patients attaining higher GAIS scores than those in Group A (Table 1).

**TABLE 1:** Sample characteristics.

	Gender	*n*	Mean age
Group A	Male	23	39.6
	Female	43	47.0
Group B	Male	16	41.7
	Female	50	46.2

**FIGURE 3:**
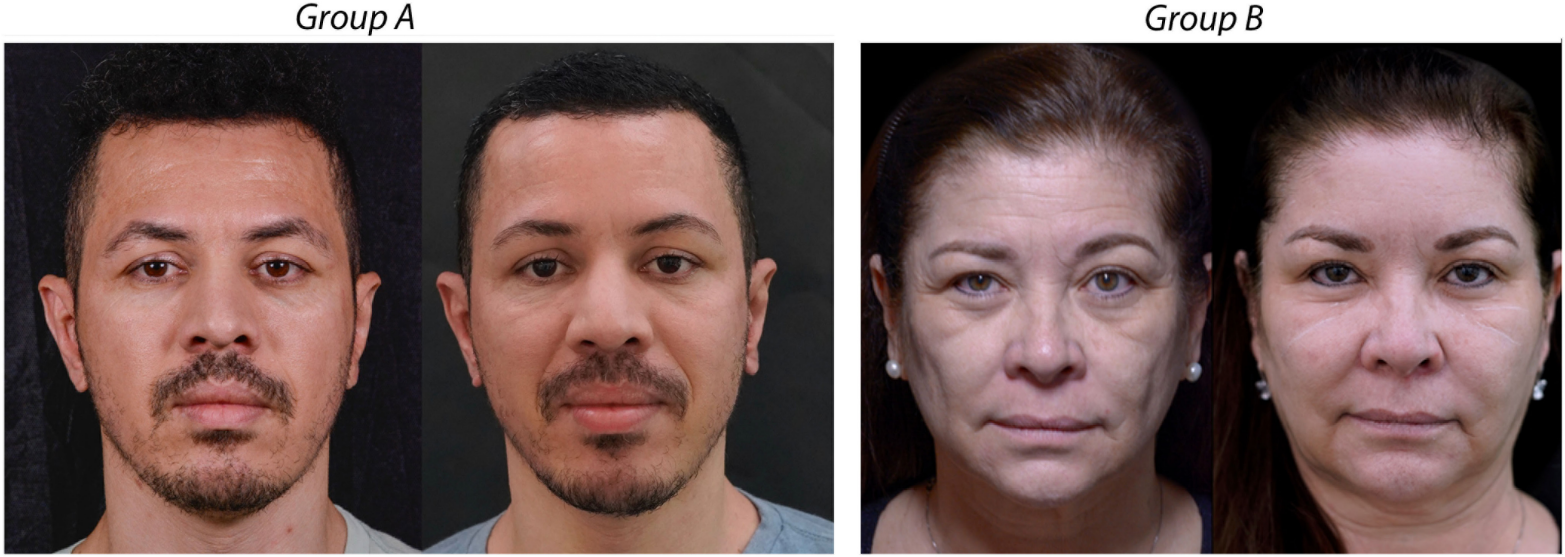
Illustrative results.

The incidence of nodules was low, totaling 3.7%. Although not statistically significant, Group A had four times more occurrences of nodules compared to Group B. In assessing the occurrence of nodules following the treatment, we observed no significant differences between the groups over 6 months (*p* = 0.36, Table 2). Specifically, within Group A, nodules were detected in four patients, primarily concentrated in the perforated region, whereas only one patient in Group B exhibited this adverse effect. Statistical disparities were evident between the groups (*p* = 0.03), with Group B demonstrating superior outcomes (Table 2). Notably, 11 patients in Group A and 3 in Group B showed no improvement in the identified abnormalities (Figure 3).

**TABLE 2:** Presence of nodules and improvement of irregularities on the forehead.

	Group A	Group B	*p*‐value
The presence of nodules on the forehead			
No	62	65	
Yes	4	1	0.36
The presence of irregularities in the forehead			
No	55	63	
Yes	11	3	**<0.04**

*Note*: Bold value denotes statistical significant difference.

## 4 DISCUSSION

HA and CaHA are the most popular dermal fillers in studies and clinical practice. HA fillers have gained popularity due to increased soft tissue, their high biocompatibility, biodegradability, fibroblast cell stimulation for collagen production, hydrophilia, water absorption and retention capacity, volume effect, and easy wrinkles correction.^24^

CaHA filler has been found effective for increasing facial soft tissue.^13^, ^25^, ^26^ CaHA is a biodegradable filler with synthetic CaHA microspheres suspended in a carrier gel. The soluble conveyor gel evenly distributes the microspheres, creating an interstitial space between the microspheres at the injection site. The gel is gradually absorbed, leaving behind the microspheres, which induce the production of new collagen around the microspheres.^27^, ^28^, ^29^ The CaHA filler's action mechanism initially involves the distribution of CaHA microspheres carried by a soluble gel. These particles remain anchored at the injected site and promote long‐term collagenases, electrogenesis, and angiogenesis, ensuring effects lasting an average of 15 months.^25^, ^30^ CaHA is an alternative to HA for volume and support on the face.^13^ It has high viscosity and elasticity^14^, ^15^ compared to HA, showing more significant tissue lifting capacity.^13^

However, the volume provided by CaHA depends on the region of the face that was deposited, the patient's response, and other individual factors, which makes it difficult to predict the volume it will provide. In contrast, the volume provided by the filler with HA is less affected by individual factors varying only with their rheological characteristics and the location of the face applied. For this reason, if the HA filler is injected with CaHA, we can gain CaHA to produce collagen and elastic fibers, in addition to the benefit of partial reversibility and proportional volume. Another advantage is maintaining volume until neocolagenesis occurs and promoting additional volume, with greater tissue integration between the filler and the tissue. Thus, we can consider improving HA's mechanical and biological properties when CaHA is added^29^ in addition to the more constant and predictable volume at the injection site.^31^

The mixture of CaHA and HA undergoes partial degradation due to the presence of HA. CaHA and HA combination allows for correcting irregularities using hyaluronidase, providing more flexibility in achieving the final result. Previous treatment with botulinum toxin prevents filler movement until CaHA promotes additional stimulation of the extracellular matrix, making the filling more stable.^32^

A pilot study tested the clinical efficacy of the combination of CaHA and HA in a single mixture and performed a biopsy in five patients, showing collagen beams without inflammation 6 months after the mixture injection.^33^ They argued that HA would compensate for the loss of volume when the gel dissipates from the CaHA microspheres before neocollagenesis. They concluded that CaHA mixed with HA filler produced stable short‐ and long‐term effects on the lower facial contour correction and determined the safety of the mixture for use.^33^ Our study found significant differences between groups in volume for correcting irregularities on the forehead with CaHA versus CaHA associated with HA after 180 days. The mixture is more effective in proportion to the volume for correcting irregularities and even better in improving the signs of aging.

The current protocol used a 3.5 mL solution, which might be insufficient to correct advanced cases of forehead irregularities, even with 1.25 mL CaHA and 1.25 mL HA. A lower volume of solution was chosen to avoid vascular compression. Consequently, severity cases may require more than two sessions to achieve an acceptable result. One of the limitations of the current study was that it evaluated only a single session.

In our study, there were no complications such as inflammation or hypersensitivity reactions, with only one case of nodule in this group being reversible. New clinical studies are needed to confirm the effectiveness and safety of the supraperiosteal technique for cheek fillers using the combination of CaHA and HA with greater filler volume.

Our study's notable limitation is its reliance on a single‐center design. Consequently, caution should be exercised in extrapolating our findings to a more heterogeneous population, and future research endeavors may benefit from incorporating multiple centers to enhance the external validity of the study's conclusions.

The use of the supraperiosteal technique for forehead filling, using a combination of CaHA and HA, proved to be more effective than the isolated use of CaHA in improving the aesthetic appearance of aging signs and frontal irregularities. Additionally, this approach led to a lower frequency of nodules, and the nodules that did occur in the group using the hybrid mixture were easily resolved.

## AUTHOR CONTRIBUTIONS

Study concepts: N.S., D.P.R., K.M.S.M., A.L.S.G., M.T.S. study design: N.S., D.P.R., A.L.S.G., M.T.S. data acquisition: N.S., D.P.R., K.M.S.M., M.T.S. quality control of data and algorithms: A.L.S.G., M.T.S. data analysis and interpretation: A.L.S.G., M.T.S. statistical analysis: K.M.S.M., A.L.S.G., manuscript editing: N.S., D.P.R., K.M.S.M. manuscript review: A.L.S.G., M.T.S. all authors have read and approved the final manuscript.

## CONFLICT OF INTEREST STATEMENT

The authors deny any conflicts related to this study.

## ETHICS STATEMENT

The clinical trial in this study (CAAE: 58145022.2.0000.5374) was examined and approved by the Sao Leopldo Mandic Faculty Ethics Committee.

## Data Availability

The data that support the findings of this study are available on request from thecorresponding author. The data are not publicly available due to privacy or ethicalrestrictions.
